# Mass coral bleaching due to unprecedented marine heatwave in Papahānaumokuākea Marine National Monument (Northwestern Hawaiian Islands)

**DOI:** 10.1371/journal.pone.0185121

**Published:** 2017-09-27

**Authors:** Courtney S. Couch, John H. R. Burns, Gang Liu, Kanoelani Steward, Tiffany Nicole Gutlay, Jean Kenyon, C. Mark Eakin, Randall K. Kosaki

**Affiliations:** 1 Hawaiʻi Institute of Marine Biology, Kāne‘ohe, Hawaiʻi, United States of America; 2 Ecosystem Sciences Division, Pacific Islands Fisheries Science Center, NOAA, Honolulu, Hawaiʻi, United States of America; 3 Coral Reef Watch, NOAA/NESDIS/STAR, College Park, Maryland, United States of America; 4 Global Science & Technology Inc., Greenbelt, Maryland, United States of America; 5 Marine Science Program University of Hawaiʻi at Hilo, Hilo Hawaiʻi, United States of America; 6 U.S. Fish and Wildlife Service, Honolulu, Hawaiʻi, United States of America; 7 Coral Reef Watch, NOAA/NESDIS/STAR, College Park, Maryland, United States of America; 8 NOAA Papahānaumokuākea Marine National Monument, Honolulu, Hawaiʻi, United States of America; Universita degli Studi di Genova, ITALY

## Abstract

2014 marked the sixth and most widespread mass bleaching event reported in the Northwestern Hawaiian Islands, home to the Papahānaumokuākea Marine National Monument (PMNM), the world’s second largest marine reserve. This event was associated with an unusual basin-scale warming in the North Pacific Ocean, with an unprecedented peak intensity of around 20°C-weeks of cumulative heat stress at Lisianksi Island. *In situ* bleaching surveys and satellite data were used to evaluate the relative importance of potential drivers of bleaching patterns in 2014, assess the subsequent morality and its effects on coral communities and 3D complexity, test for signs of regional acclimation, and investigate long-term change in heat stress in PMNM. Surveys conducted at four island/atoll (French Frigate Shoals, Lisianski Island, Pearl and Hermes Atoll, and Midway Atoll) showed that in 2014, percent bleaching varied considerably between islands/atolls and habitats (back reef/fore reef and depth), and was up to 91% in shallow habitats at Lisianski. The percent bleaching during the 2014 event was best explained by a combination of duration of heat stress measured by Coral Reef Watch’s satellite Degree Heating Week, relative community susceptibility (bleaching susceptibility score of each taxon * the taxon’s abundance relative to the total number of colonies), depth and region. Mean coral cover at permanent Lisianski monitoring sites decreased by 68% due to severe losses of *Montipora dilatata* complex, resulting in rapid reductions in habitat complexity. Spatial distribution of the 2014 bleaching was significantly different from the 2002 and 2004 bleaching events likely due to a combination of differences in heat stress and local acclimatization. Historical satellite data demonstrated heat stress in 2014 was unlike any previous event and that the exposure of corals to the bleaching-level heat stress has increased significantly in the northern PMNM since 1982, highlighting the increasing threat of climate change to reefs.

## Introduction

Coral bleaching involves the breakdown of the symbiosis between a coral and its endosymbionts (*Symbiodinium* spp.) in response to environmental stress (such as anomalous changes in temperature [1–3], salinity [4], sedimentation [5], and/or light [6], resulting in the expulsion of the algae [7]. Under severe stress, due to the loss of a significant amount of symbionts and/or photosynthetic pigments in the symbionts, the coral exhibits a paled or “bleached” appearance. Hermatypic corals rely on their algal symbionts for photosynthetic products that typically supply a majority of their energetic requirements [8, 9]. Consequently, if the stress does not abate and the symbiont community is not reestablished mortality can occur [10]. The global rise in widespread (mass) bleaching events has been linked definitively to increasing ocean temperatures [1, 4, 11, 12].

Mass bleaching events and associated mortality can dramatically alter ecosystem structure and function. In the short-term, heat stress, with or without bleaching, renders corals more susceptible to disease [13, 14] and can impede reproduction [15, 16] and growth [17, 18]. As ecosystem engineers, corals provide both biological and physical structural complexity that supports high levels of diversity and organismal abundance by providing a large array of microhabitat types. Bleaching-induced mortality can result in widespread loss of coral cover and shifts in coral community structure [19–21]. In addition, reduced carbonate accretion by bleached corals combined with bioerosion can also dramatically reduce reef growth and 3D structural complexity [21–25] and ultimately make reefs more susceptible to other large scale disturbances [26, 27]. These declines in structural complexity have been linked to reduced abundance and diversity of reef fish through the removal of habitat, decoupling organismal relationships that drive critical ecological processes [28–30]. While there is growing evidence of the impacts of bleaching on reef complexity, conventional methods lack the resolution to accurately quantify changes in reef structure associated with coral mortality and thus may be underestimating the speed at which structural changes are occurring. It is critical to apply techniques capable of monitoring 3D reef complexity to determine how coral mortality affects habitat dynamics and larger ecological processes.

Mass coral bleaching events and exposure to heat stress have increased in frequency, severity and geographic extent since the 1980s [1, 4, 11, 12, 31], presenting a major threat to reef ecosystems with up to 90% of reefs predicted to experience severe annual bleaching by 2055 [32]. To date there have been three documented global mass bleaching events. While bleaching was potentially global in 1983 [33], the first well-documented global event occurred in 1998 and resulted in widespread mortality of the world’s reefs and as much as 80% mortality of live coral in portions of the Indian Ocean [10]. In 2010 global bleaching spanned from the western Indian Ocean to the Caribbean and was particularly devastating in Southeast Asia [34, 35]. The most recent global bleaching event began in Guam and the Commonwealth of the Northern Mariana Islands in June 2014 and encompassed Hawaiʻi, Marshall Islands, and Florida during 2014. It continued into 2015, moving into the South Pacific, Indian Ocean, central and eastern tropical Pacific, and then Caribbean, making it a truly global event [36]. As the 2015–2016 El Niño developed, reaching its peak strength in November-December 2015, the bleaching in 2016 spread across the Pacific and Indian Oceans [Vargas-Ángel pers. comm., 11]. As of June 2017, this was the longest, most widespread, and most severe global bleaching event on record with over half of reef areas, including Hawaiʻi and the Great Barrier Reef, experiencing multiple mass bleaching events during this period [65, 11].

Rising ocean temperatures caused by anthropogenic climate change have increased significantly since the 1980s, with the hottest global ocean temperatures on record occurring in 2014, 2015, and 2016 (NOAA-NCEI annual climate report, https://www.ncdc.noaa.gov/sotc/global), bringing corals close to or above their thermal tolerance threshold [2, 3, 7, 12, 37]. Mass bleaching usually occurs during prolonged exposure (typically four to six weeks) to elevated temperatures 1°C above the local average temperature of the warmest month of the year [16, 38], with severity increasing with both length and magnitude of exposure [7]. However, the degree to which corals at a given reef bleach can vary considerably and is determined by factors other than just heat stress, such as local hydrodynamics, UV irradiance and depth [2, 39–42], and thermal history [34, 43–46]. Species and coral communities also vary markedly in their ability to resist and recover from heat stress with some species and communities unable to withstand novel or repeated heat stress and others demonstrating capacity to acclimatize [19, 20, 34, 42, 46–48]. For example, mass bleaching in Southeast Asia during 2010 was less severe in regions that bleached during 1998 and had greater historical temperature variability [34]. While physiological assessments and manipulative studies are needed to explicitly test for acclimation, comparing recent bleaching levels to standardized historical bleaching can provide early signs of acclimation thus helping to identify resilient populations and communities.

Despite its higher tropical/subtropical latitude, the Hawaiian Archipelago has not escaped regional heat stress and the effects of mass bleaching. In fact, Jokiel and Coles [41] predicted the impending onset of mass bleaching in Hawaiʻi based on rising temperatures nearing upper thermal tolerance. In 1996, the first recorded bleaching primarily affected Kāneʻohe Bay, Oʻahu and other northern Main Hawaiian Island reefs [2]. In 2002 and 2004 the northern atolls in the Papahānaumokuākea Marine National Monument (PMNM, encompassing the Northwestern Hawaiian Islands) experienced severe bleaching [49]. Low to moderate bleaching has also been recorded in these northern atolls in 2007, 2009 and 2010 (NOAA Pacific Islands Fisheries Science Center Ecosystem Sciences Division pers. comm.). In 2014, PMNM experienced widespread and severe bleaching across the central and northern PMNM that also reached the Main Hawaiian Islands [50, 51].

The unprecedented heat stress in PMNM during 2014 and repeated heat stress and regional bleaching since the early 2000s provided an opportunity to better understand the factors driving bleaching, its impacts on these subtropical coral communities and changing thermal conditions in PMNM. In this study, we aimed to: 1) assess the long-term trend of heat stress in PMNM; 2) test for potential signs of acclimation by examining the thermal history across several regions and corresponding temporal change in bleaching levels and community composition during three major bleaching events, 3) link patterns in the proportion of bleached corals in 2014 (% bleaching) to the heat stress measured by NOAA Coral Reef Watch’s satellite-based heat stress products, depth, and coral community bleaching susceptibility; and 4) assess consequences of the 2014 event on the composition and 3D structure of coral communities.

## Methods

### Ethics statement

This study was conducted in the fully protected conservation area Papahānaumokuākea Marine National Monument (PMNM), which was designated as a US National Monument Presidential Proclamation 8031 on June 15, 2006 under the authority of the Antiquities Act (16 U.S.C. 431–433). Entry into and research in PMNM requires a research permit issued by NOAA/PMNM and approved by NOAA, the U.S. Fish and Wildlife Service and the State of Hawaiʻi. Research in this study was conducted under the following permits: PMNM-2014-12 to C. Couch, PMNM-2014-018 to S. Godwin, PMNM-2014-005 to LCDR Daniel Simon, and PMNM-2015-13 to C. Couch. This study did not involve contact or interactions with endangered or protected species.

### Bleaching heat stress analysis

NOAA Coral Reef Watch (CRW) produces both near real-time and historical satellite sea surface temperature (SST)-based global coral bleaching heat stress monitoring products (https://coralreefwatch.noaa.gov). Our study used two CRW product suites: CRW’s daily 5 km global near real-time monitoring product suite version 3 (5kmv3), which is an update (M. Eakin and G. Liu, personal communication) of the version 2 described in Liu et al. (2014); and a historical 1982–2016 daily 25 km global product suite produced by CRW (G. Liu, personal communication) based on NOAA’s daily Optimum Interpolation Sea Surface Temperature version 2 (dOISST.v2; [52]). The 5kmv3 product was derived from NOAA/National Environmental Satellite, Data, and Information Service’s (NESDIS) operational daily 5 km global Geo-Polar Blended SST Analysis. Both products suites were based on the same algorithm described by Liu et al. [39], but the 5kmv3 used a new climatology developed from recently reprocessed 5 km Geo-Polar Blended SST back to 2002 and the reprocessed Operational Sea Surface Temperature and Sea Ice Analysis back to 1985 [53, 54] while the OISST data used a climatology developed directly from its own data. Annual composites of daily products from both product suites were also provided by CRW. CRW’s SST and Degree Heating Week (DHW) time series were extracted from both product suites for select PMNM reef/atoll locations to assess the heat stress in PMNM.

CRW’s DHW product provides a measure of accumulated heat stress used to predict bleaching [37]. In general, DHW ≥ 4°C-weeks is associated with significant bleaching and categorized by CRW as Alert Level 1; DHW ≥ 8°C-weeks is associated with widespread bleaching and significant mortality and categorized as Alert Level 2. DHW is calculated as an accumulation of daily Coral Bleaching HotSpots over consecutive 12 weeks for the days when HotSpot values are at least 1°C. CRW’s definition of HotSpot is the positive SST anomaly above the Maximum of the Monthly Mean SST climatology (known as MMM), generally the upper thermal tolerance level for corals. CRW’s algorithm was described in detail in Liu et al [39].

The high-resolution CRW 5 km dataset for 2014–2015 was used for in-depth investigation of the 2014 and 2015 mass bleaching events in PMNM, while CRW’s 25 km OISST dataset was used to assess long-term change in SST and heat stress in PMNM over a 34-year period of 1982–2016.

For the OISST dataset, there are 11 pixels across the four islands/atolls where bleaching assessments were conducted in this study and 16 pixels that cover all of the shallow reefs in PMNM. DHW values derived from dOISST.v2 are slightly higher than those from the CRW 5km product. This results in part from the coarser spatial resolution of the OISST (25km) that picks up anomalies over a wider area, and from a slightly warmer bias in the OISST dataset. Values from the two datasets are not compared against each other in the analyses.

### Bleaching surveys

**Fig 1:**
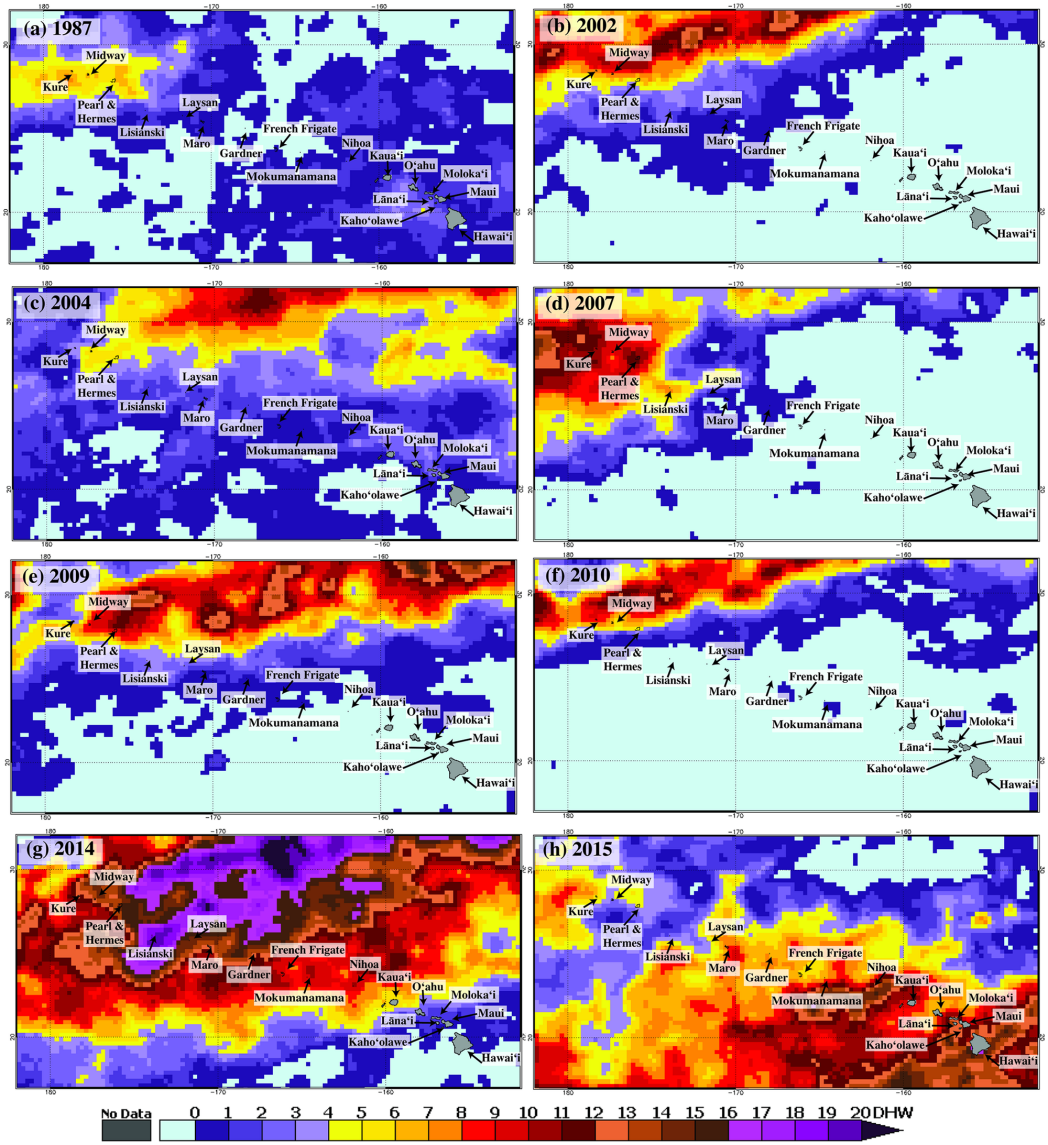
Annual maximum composite of CRW’s dOISST.v2 (25km) Degree Heating Weeks, across the Hawaiian Archipelago for years (a) 1987, (b) 2002, (c) 2004, (d) 2007, (e) 2009, (f) 2010, (g) 2014, and (h) 2015), when DHW ≥ 4°C-weeks (the minimum level associated with significant bleaching) appeared in PMNM.

To document the spatial extent of bleaching in 2014, we used two approaches. We used a stratified-random design employed by NOAA PMNM and NOAA PIFSC’s Ecosystem Sciences Division. Bleaching assessments were conducted between August 14^th^ and August 28^th^, 2014 at French Frigate Shoals (FFS), Lisianski Island (LIS), Pearl and Hermes Atoll (PHR), and Midway Atoll (MID) as part of NOAA’s annual Reef Assessment and Monitoring Program (NOAA cruise HA-14-04; Permit #: 2014–012) (Fig 1). The atolls/islands (regions) surveyed in this study were preselected based on available time and concurrent research priorities. In each region, randomly-selected hard-bottom sites were allocated into habitat strata based on each stratum’s proportional area and the variance structure of the population within the stratum [55]. Where and when possible, we surveyed the following habitat types: shallow fore reef (0–6 m), moderate fore reef (6–18 m), deep fore reef (18–30 m), shallow back reef (0–6 m), and moderate back reef (6–18 m). All habitat categories were not surveyed at all regions due to lack of certain habitats or logistical constraints. Ten to 13 sites were surveyed at each region and habitat, and the number varied depending on the proportion of each habitat area present. Within each randomly-selected site, 2 to 4 belt transects were haphazardly placed on the reef. Adult coral colonies (≥ 5 cm) were surveyed within four (1.0 x 2.5 m) segments on each transect with one meter gaps between segments to better capture spatial heterogeneity on the reef. Segments were pooled for each transect (10m^2^ per transect and a total of 20m^2^ to 40m^2^ per site). Data collected on each colony included: species, health state (apparently healthy or bleached), and % of pigmentation lost to the nearest 5%. We present bleaching data as % bleached, which is the proportion of colonies along a transect that were severely bleached (>50% of colony fully bleached or the entire colony showed a 50% reduction in pigmentation).

To increase the spatial extent of our surveys in 2014 and assess the temporal patterns in bleaching and mortality at FFS, LIS, PHR, and MID in 2014 and 2015, we also surveyed permanent transects that did not overlap with stratified-random sites described above. These surveys were conducted between September 11^th^ and 26^th^, 2014 on NOAA cruise HA-14-05, and resurveyed between August 2^nd^ and 20^th^, 2015 on NOAA cruise HA-15-06 (Permit #: 2015–013). At each region, we surveyed sites across shallow and moderate depths in the fore reefs and back reefs when weather conditions and time permitted. Due to time constraints, we only surveyed shallow back reef sites at MID. At each site, we located existing permanent pins or established new pins. We conducted three 10 x 1m belt transects with 10 m between transects. Along each transect we assessed bleaching as described above.

To compare 2014 bleaching levels to prior recorded events (2002 and 2004), we used historical bleaching data (see [49] for details). In 2002 and 2004, bleaching surveys were conducted along one or two 25 x 2m belt transects at 66 permanent sites across PMNM. Colonies were identified to genus or species in 2002 and species in 2004 with bleaching recorded if more than half of its live tissue had lost at least 75% of its normal pigmentation. To compare bleaching levels in 2002 and 2004 to 2014, we only analyzed data for sites visited in all three years (14 sites total) and pooled replicate transects together for each site and year. In all three years % bleaching was defined as the proportion of colonies that had lost at least 75% of its pigmentation. While low to moderate bleaching has been observed in 2007, 2009, and 2010, we did not have enough permanent sites that overlapped with 2002, 2004, and 2014 to include in our analyses. CRW’s historical 25 km satellite DHW data for 2002, 2004, and 2014 were used to compare stress pattern of 2014 with those of 2002 and 2004.

### Coral cover surveys

To assess the impacts of the 2014 bleaching event on coral communities, we conducted photo quadrats at 1m intervals, 1m above the substrate using a Canon G15 (11 total frames per transect) to assess changes in coral cover. Each photograph was analyzed in Coral Point Count program with Excel extension (CPCe) [56]. Thirty random points were overlaid on each photograph, and the benthic component under each point was identified to the lowest possible taxonomic level. To reduce observer variability, all photographs were processed by a single individual. The raw point data from all photographs on a transect line were combined to calculate the percent cover of each benthic component for the entire belt transect. Comparable coral cover data were not available for 2002 or 2004 and therefore were not included in our study.

### 3D structural surveys

To quantify the structural changes to the reef as a result of the 2014 heat stress event, images were collected at the most severely bleached site (LIS-4067) within a 10x3m plot in 2014 and 2015. Ground control points were placed at the corners of the plot to enable accurate geo-referencing and spatial scaling of the survey plot. Images of the survey area were collected from planar and oblique angles with 70–80% overlap. All photos were taken with a Canon 5D Mark III digital SLR camera with a 24-70mm lens (Canon USA Inc., New York, USA) in an Ikelite housing with an 8-inch hemispheric dome port (Ikelite Underwater Systems, Indianapolis, USA). Three-dimensional (3D) reconstructions of the coral reef plot were rendered using Agisoft modeling software (Agisoft LLC., St. Petersburg, Russia). Protocols and settings were used based on methods designed for creating high-resolution 3D models of underwater coral reef habitats, and the resulting models were integrated into geospatial software to quantify 3D structural metrics [57]. Two-dimensional orthophoto mosaics were imported into CoralNet software to compute the percent cover of live coral, dead coral, and macroalgae. Alignment of the 2014 and 2015 3D reef point clouds were completed using CloudCompare (v. 2.6.1, 2015) and the Multiscale Model to Model Cloud Comparison (M3C2) algorithm to quantify the exact 3D structural and volumetric change in the coral reef substrate associated with the bleaching event [29]. Volumetric change was calculated using CloudCompare to align and register the 3D points clouds from each time point in order to compare 3D changes over time. The M3C2 algorithm detected statistically significant deviation in the x,y,z location of the point clouds over time to assess changes in the 3D structure. The area of loss or gain between the two point clouds was then computed to determine volumetric increase/decrease between the time points of the surveys. This analysis enabled quantification of how much the volume of the point cloud increased or decreased over time.

### Statistical analyses

All bleaching and coral cover data were analyzed in R version 3.2.3. All bleaching, coral cover, heat stress and site metadata are included in S1 File and 3D metrics are included in S2 File. To assess long-term change in heat stress since the 1980s, CRW’s annual maximum composites of daily 25 km DHW for each individual year and pixel were used. The 16 shallow water reef pixels across PMNM were grouped into three general regions: South (Nihoa, Mokumanamana, French Frigate Shoals), Middle (Maro Reef, Laysan Island, Lisianski Island), and North (Pearl and Hermes Atoll, Midway Atoll, and Kure Atoll) with 1–3 pixels/island or atoll. To identify trends for each general region, we ran Mann-Kendall nonparametric trend tests with Sen’s slope estimates on the annual medians of the pixel annual maximum DHW for each of the three general regions.

To compare % bleaching in 2014 to previous bleaching events we conducted three separate analyses on 14 permanent sites that were surveyed in 2002, 2004 and 2014. First, to determine whether bleaching varied significantly among years, we used separate generalized linear mixed models (GLMMs) with year treated as a fixed effect and site as a random effect and used generalized linear hypothesis tests (glht package) to test for differences among years at each region. We then used general linear models (GLMs) with binomial errors and logit links to assess the relationship between % bleaching in 2002, 2004, and 2014 and heat stress during the time of the survey. To test for signs of acclimation, we extracted the residuals from the binomial regression of % bleaching and heat stress (S1 Fig) then used the residuals to test whether bleaching irrespective of heat stress varied significantly among years using separate ANOVAs and tukeyHSD post hoc tests for each region. Midway could not be included in analyses as only two sites were surveyed in all three years. To test whether coral communities shifted between years at the regions where we saw a significant change in bleaching, we used a distance-based permutational multivariate analysis of variance (PERMANOVA) on a Bray—Curtis similarity matrix of all species or genera and explored data visually using a non-metric multidimensional scaling (NMDS) scatter plot with Bray-Curtis distance. Coral cover information was unavailable for 2002 and 2004, so each taxon was expressed as the % of total coral community. In 2002, corals were identified to species or genus, but corals were identified to species in 2004 and 2014. To ensure comparability between years, we applied the taxonomic resolution used in 2002 to all years.

To identify the potential drivers of % bleaching (% of colonies that bleached > 50%) in 2014 and maximize the spatial extent of our surveys, we combined our 2014 stratified-random and permanent transect datasets. We used GLMMs with binomial errors and logit links using maximum likelihood estimation (glmer function in lme4 package). We constructed 21 hierarchical and single-factor models with site as random effects and region, 2014 heat stress, depth, and relative community bleaching susceptibility as fixed effects. Given the potential link between increased temperature variability and increased bleaching resistance [43–45], we also used the dOISST.v2 dataset to calculate SST variability using several metrics described by Heron et al. [31] and Guest et al. [34]. However, we lacked enough within-region SST variability to include these in the models. For 2014, heat stress we used the CRW 5 km DHW data at the time of the survey. A region by heat stress interaction term was also included in the models given the variability in regional responses to heat stress. Due to the subjectivity in habitat classification, the unbalanced coverage of habitat types across the four regions, and the collinearity of habitat and relative susceptibility but potential differences in bleaching with depth [58, 59], we substituted depth for habitat in our models. To calculate coral community bleaching susceptibility we expanded upon the % bleaching resistance metric described by Manzello et al. [60]. All coral species were scored from least (1) to most susceptible (10) to bleaching using a combination of species bleaching levels in 2014 (S3 Table), 2004 [49] and unpublished data from NOAA PIFSC’s Ecosystem Sciences Division. Relative community bleaching susceptibility CS for a given site was calculated as follows:

$$CS_{s}=\;\left(\sum_{t=1}^{T_{s}}A_{ts}S_{t}\right)/A_{s}$$

where, taxon is indexed with subscript *t*, T_*s*_ is the total number of taxa for a given site *s*, A_*ts*_ is the abundance a given taxon at a given site, S_*t*_ is the susceptibility score of a given taxon and A_*s*_ is the abundance of all taxa at a given site. A region x susceptibility score interaction term was also included in the models due to the regional variability in community structure and bleaching susceptibility. We conducted model selection (AICcmodavg package) using an information theoretic approach, comparing corrected Akaike’s information criterion (AICc, ΔAICc and AIC weight *Wt*) to rank and determine which variable(s) best fit bleaching [61]. *Wt* range from 0 to 1, with the ‘best-fit’ models having the highest weight and lowest ΔAICc. Models with ΔAICc < 10 were considered most reasonable [62].

To determine whether percent coral cover declined significantly between 2014 and 2015 and varied between regions, we used linear mixed models (LMM) with LRTs following tests for normality and equal variances. The interaction of year and region were treated as fixed effects and site nested within year was treated as random effects. To determine which regions experienced significant changes in coral cover, we used separate LMMs for each region with Bonferroni corrections at α = 0.05. To determine whether % coral cover for each species (>1% cover) changed significantly between 2014 and 2015, we used Mann Whitney U tests with Bonferroni corrections for each region. We conducted separate linear regressions for each region (with Bonferroni corrections) to assess whether proportional change in overall coral cover (2014 coral cover—2015 coral cover/2014 coral cover) was correlated with 2014 bleaching incidence. We used a linear regression to determine whether % coral loss was related to maximum heat stress in 2014.

Mean values of the 3D structural metrics, coral cover, and coral diversity were compared using two sample t-tests in order to determine if community composition and 3D habitat complexity were significantly affected (α = 0.05) by the bleaching event at the LIS-4067 site. The M3C2 algorithm derived distance measurements between the 2014 and 2015 point clouds in order to statistically examine volumetric changes with a 95% LOD. LOD corresponds to the minimum detectable change, taking into account point cloud roughness and level of registration errors.

## Results

### Long-term change in heat stress and historical bleaching patterns

**Fig 2:**
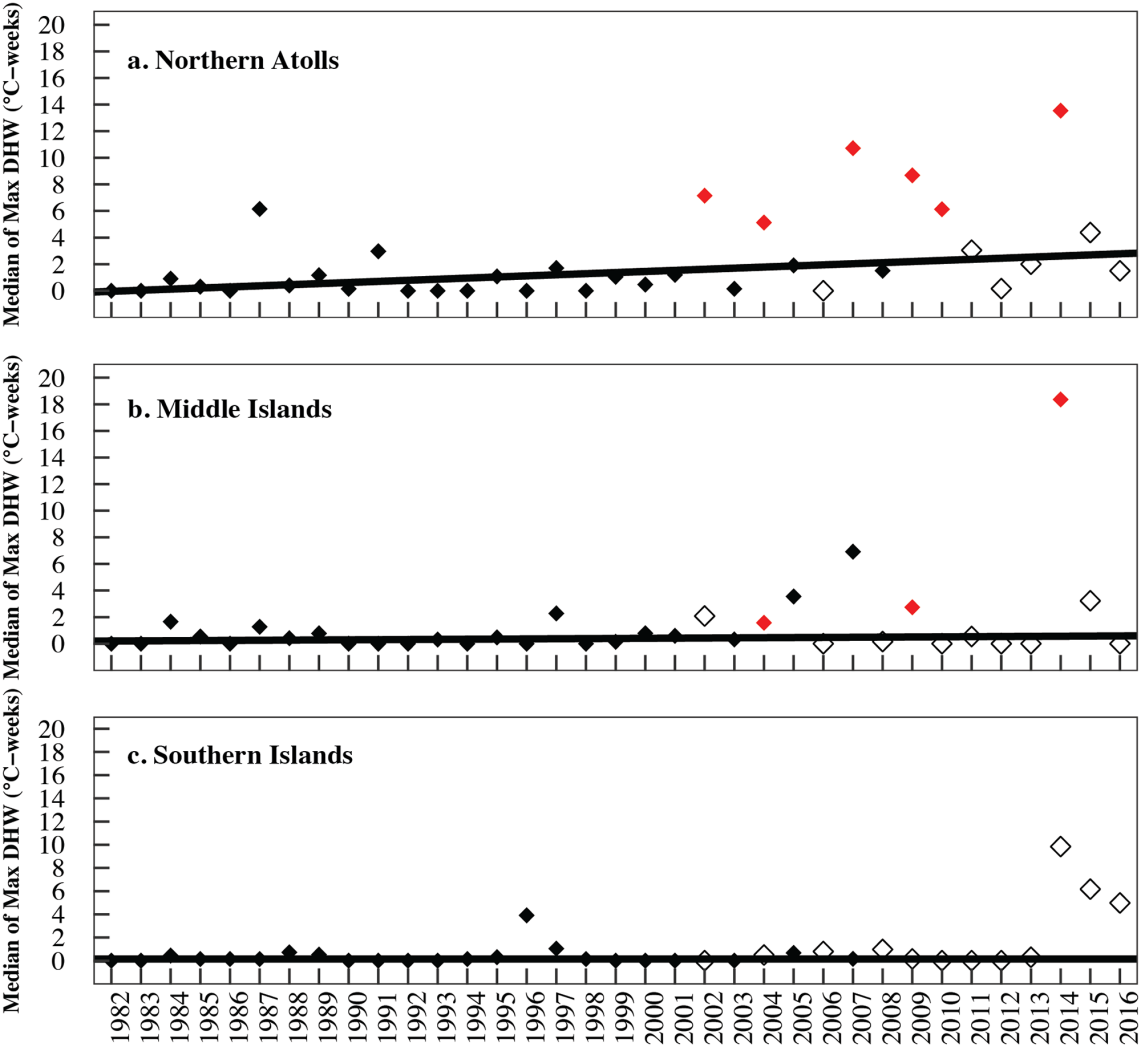
Median of annual maximum Degree Heating Weeks for pixels between 1982 and 2016 in the (a) southern islands (Nihoa (n = 1 pixel), Mokumanamana (n = 1 pixel), French Frigate Shoals (n = 3 pixels), (b) middle islands (Maro Reef (n = 1 pixel), Laysan Island (n = 1 pixel), Lisianski Island (n = 4 pixels) and (c) northern atolls (Pearl and Hermes (n = 2 pixels), Midway (n = 2 pixels) and Kure (n = 1 pixel). Lines represent Sen’s slope estimates. Data derived from CRW’s 25 km dOISST.v2-based dataset. Northern atolls: Mann-Kendell, tau = 0.366, *p* = 0.0018, Sen’s slope = 0.074; middle islands: Mann-Kendell, tau = 0.15, *p* = 0.1956, Sen’s slope = 0; southern islands Mann-Kendell, tau = 0.148, *p* = 0.205, Sen’s slope = 0.

Our results reveal that the oceanographic features of 2014–15 resulted in heat stress in 2014 and 2015 were far more extensive and severe than previous years, setting these years apart as unique in the satellite record (Fig 1). In the eight years that experienced bleaching-level heat stress since the 1980s, heat stress was primarily restricted to the northern portions of PMNM until 2014 (Fig 1). The frequency and severity of heat stress has increased significantly in the northern atolls since 1982, while heat stress has not increased significantly in the middle and southern islands (Fig 2). The trend of increasing heat stress is still significant in the northern atolls even after excluding 2014 and 2015 (Mann-Kendell, tau = 0.325, *p* = 0.008, Sen’s slope = 0.063).

**Fig 3:**
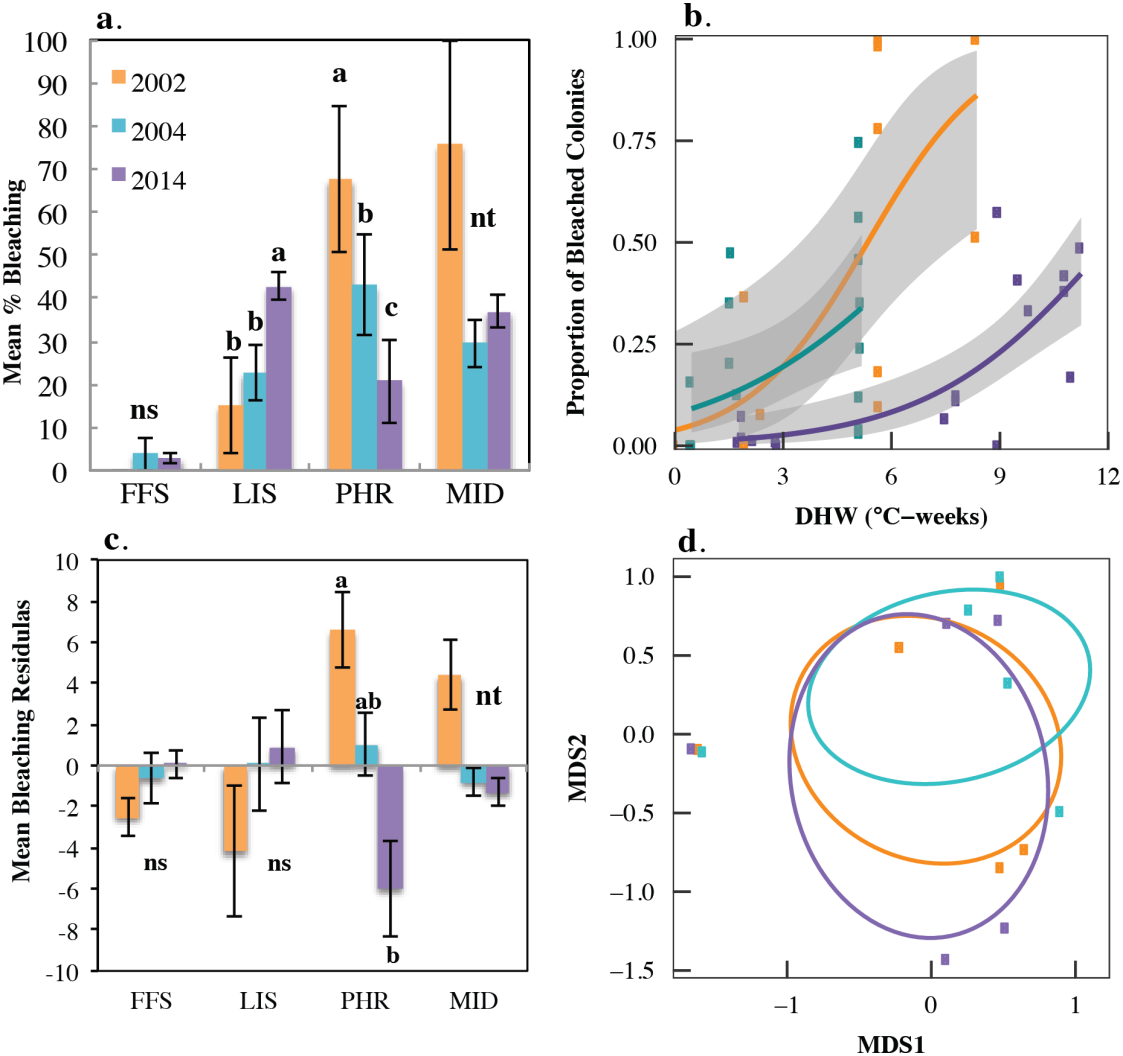
(a) Mean ± SE % bleaching at permanent study sites at French Frigate Shoals (FFS), Lisianksi Island (LIS), Pearl and Hermes Atoll (PHR) and Midway Atoll (MID) in 2002, 2004 and 2014. See S2 Table for survey dates. Letters indicate glht posthoc tests with Bonferroni Correction α = 0.016. N = 4 sites at FFS, 3 sites at LIS, 5 sites at PHR and 2 sites at MID. (b) Generalized linear regressions (binomial errors) of relationship between % bleaching and CRW’s 25 km Degree Heating Week at the time of the survey for 2002, 2004 and 2014. Solid lines: predicted bleaching (with binomial errors). Grey area: upper and lower 95% confidence intervals. (2002: R^2^ = 0.3081, p<0.0001; 2004: R^2^ = 0.2325, p<0.0001; 2014: R^2^ = 0.6979, p<0.0001). (c) Mean ± SE residuals from % bleaching vs. DHW GLM (S1 Fig) across regions and years. Letters indicate glht posthoc tests with Bonferroni Correction α = 0.016. (d) Nonmetric multidimensional scaling (nMDS) plot of coral community (% of colonies comprised of different coral taxa) differences clustered by year at Pearl and Hermes Atoll (stress = 0.057). Other regions not included because bleaching did not differ significantly between years after correcting for differences in heat stress or site-level replication was too low.

When comparing 14 permanent sites surveyed in 2002, 2004 and 2014, several patterns emerge. Bleaching incidence varied significantly between the three recorded bleaching events at LIS (LRT: χ^2^ = 73.09, df = 2, p<0.0001) and PHR (LRT: χ^2^ = 391.1, df = 2, p<0.0001) (Fig 3a). Bleaching at LIS was highest in 2014, but bleaching at PHR and MID were highest in 2002 (Fig 3a). Percent bleaching was moderately to strongly positively correlated with DHW in all three years (Fig 3b). While the slopes of the relationship between bleaching and DHW did not vary significantly between years, in 2002 corals bleached significantly more and at lower heat stress levels than 2014. (Fig 3b, S1 Table). After controlling for the influence of heat stress, mean bleaching did not vary significantly between years for FFS or LIS, but corals at PHR did bleach significantly less than expected given heat stress in 2014 compared to 2002 (Fig 3c). MID showed a similar pattern to PHR, but could not be analyzed. At PHR, the coral community structure did not change significantly between the three years (PERMANOVA, df = 2, MS = 0.076, Pseudo-F = 0.246, p = 0.8399), Fig 3d).

### Chronology of 2014 and 2015 heat stress events

**Fig 4:**
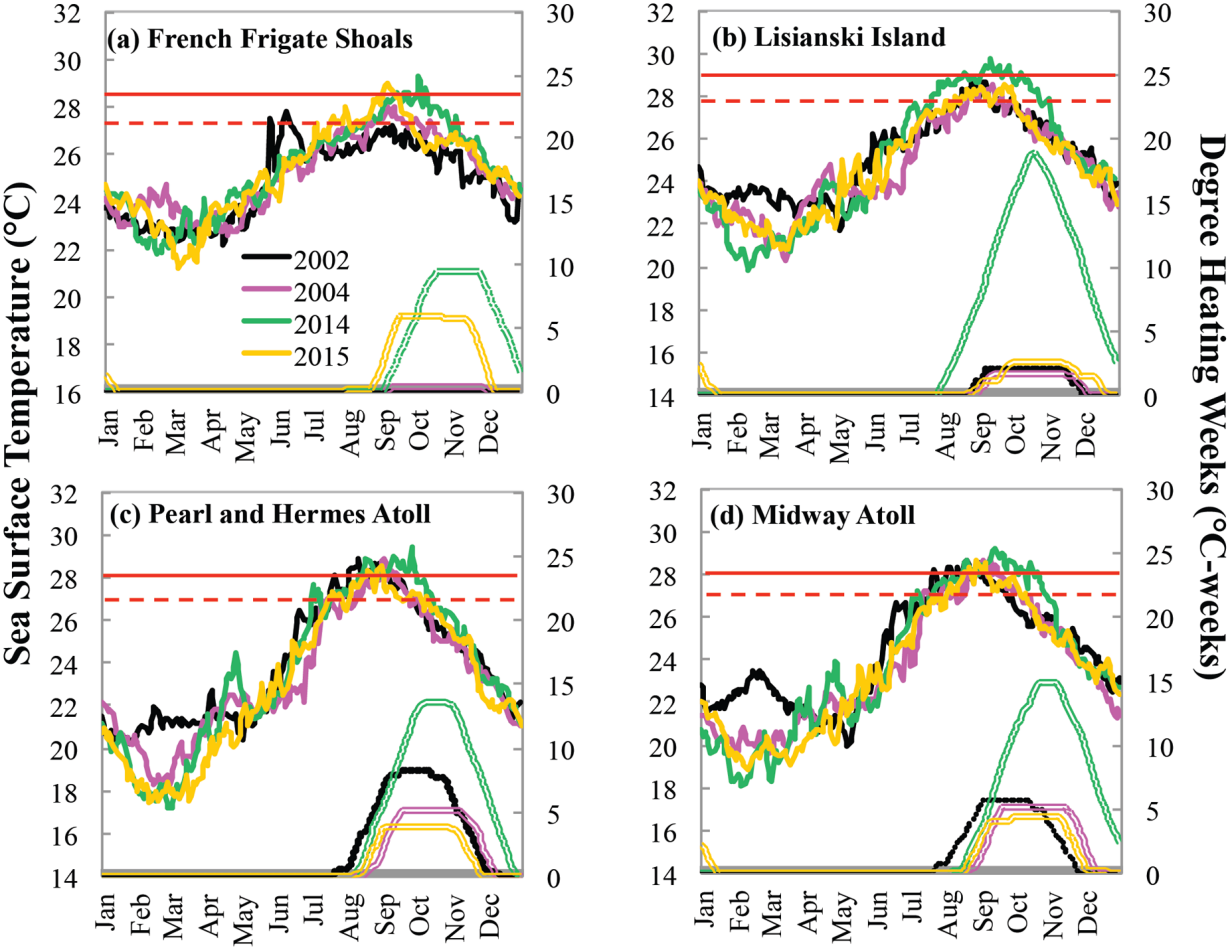
The dOISST.v2 sea surface temperature (non-red solid lines) and CRW’s dOISSTv.2-based Degree Heating Weeks (DHW, double lines) in 2002, 2004, 2014 and 2015 for (a) French Frigate Shoals, (b) Lisianski Island, (c) Pearl and Hermes Atoll, and (d) Midway Atoll. Dashed horizontal red line: the maximum of monthly mean SST climatology (MMM); Solid horizontal red line: bleaching threshold SST (1°C above MMM).

During July 2014, gradual warming began in the North Pacific extending into PMNM and by late July heat stress had accumulated in portions of PMNM, leading CRW to issue a bleaching warning (Fig 4). Warming increased rapidly in the central PMNM and by late August DHW exceeded 7°C-weeks surrounding LIS.

Heat stress continued to intensify and extended into the northern atolls (PHR and MID) reaching CRW’s Bleaching Alert Level 1 by early September (Fig 4c and 4d). At the southern extent of PMNM, heat stress reached Bleaching Alert Level 1 by late September surrounding FFS (Fig 4a), then continued into the Main Hawaiian Islands. SST did not begin to decline in PMNM until early to middle October. By the end of 2014, LIS experienced the longest accumulation of heat stress with an average maximum of 19.69°C-weeks with all other regions reaching Bleaching Alert Level 2 (Figs 1e and 4b, S2 Table).

In 2015, unlike 2014, warmer water initiated south and east of the Main Hawaiian Islands with the anomalously warm water then extending into the Main Hawaiian Islands and eventually into the southeastern portion of the PMNM. Southerly PMNM reached bleaching-level stress by late September (Fig 4a), but cumulative heat stress did not exceed 6°C-weeks in PMNM, while regions of the Main Hawaiian Islands experienced greater than 18°C weeks (Fig 1f).

### 2014 spatial and species-level patterns in bleaching

**Fig 5:**
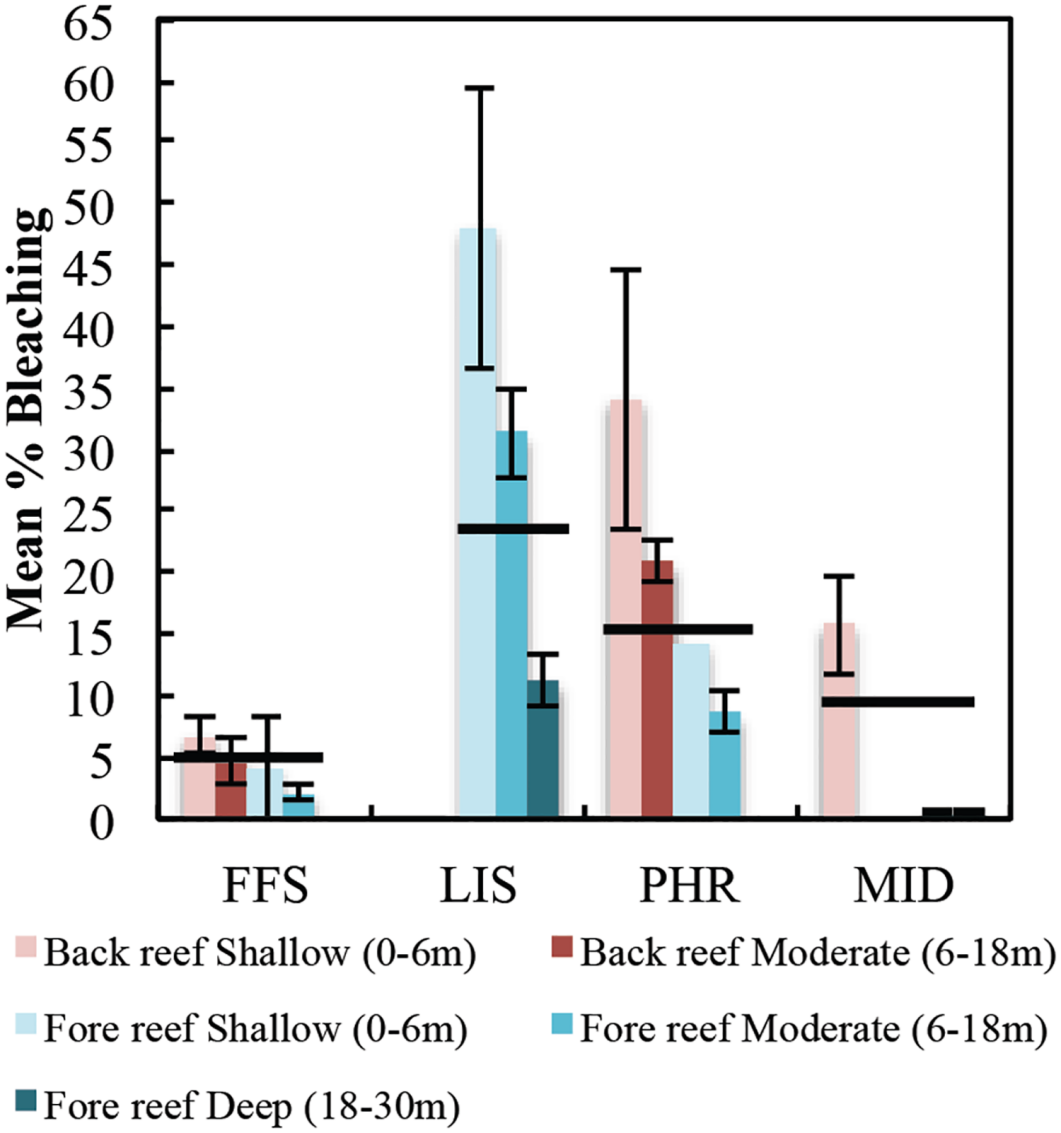
Mean ± SE % bleaching at all study sites at French Frigate Shoals (FFS), Lisianksi Island (LIS), Pearl and Hermes Atoll (PHR) and Midway Atoll (MID) surveyed between August 14^th^ to September 26^th^, 2014. Data are averaged across all sites for a regional average (black horizontal lines) and for each habitat. N = 5–27 transects/habitat/region.

Mean % bleaching during the early to middle stages of the 2014 bleaching event varied considerably across the four study regions with LIS having the highest average % bleaching (33.24 ± 4.15%), followed by PHR (15.70 ± 2.55%), MID (9.14 ± 2.54%) and FFS (5.14 ± 0.97%) (Fig 5). Within each region, bleaching also varied across habitats with shallow habitats most affected (Fig 5), with the most severe bleaching along the eastern coast of LIS at 3-4m depth where 91% of the colonies bleached by September 2014.

During 2014, 19 of the 24 coral species identified were affected by bleaching with *Montipora* spp., especially *M*. *dilatata/turgesens/flabellata* (henceforth, *M*. *dilatata* complex [63]) being most affected (S3 Table). However, the species-level susceptibility varied considerably between regions (S3 Fig). At FFS, PHR and MID, *Montipora* and to a lesser degree *Pocillopora* were the only taxa to show severe bleaching, whereas at LIS over 15% of all the dominant taxa including *Montipora*, *Pocillopora* and *Porites* bleached (S3 Fig).

Overall, % bleaching at permanent sites was up to six times higher in September 2014 compared to August 2015 (GLMM: χ^2^ = 19.7, df = 6, *p* = 0.003) (S4 Fig), which coincided with lower heat stress in 2015 (S2 Table). While 2015 surveys were conducted prior to the onset of heat stress, 2015 maximum DHW did not exceed 6.08°C-weeks (S2 Table).

### Heat stress and coral community drivers of 2014 bleaching

**Fig 6:**
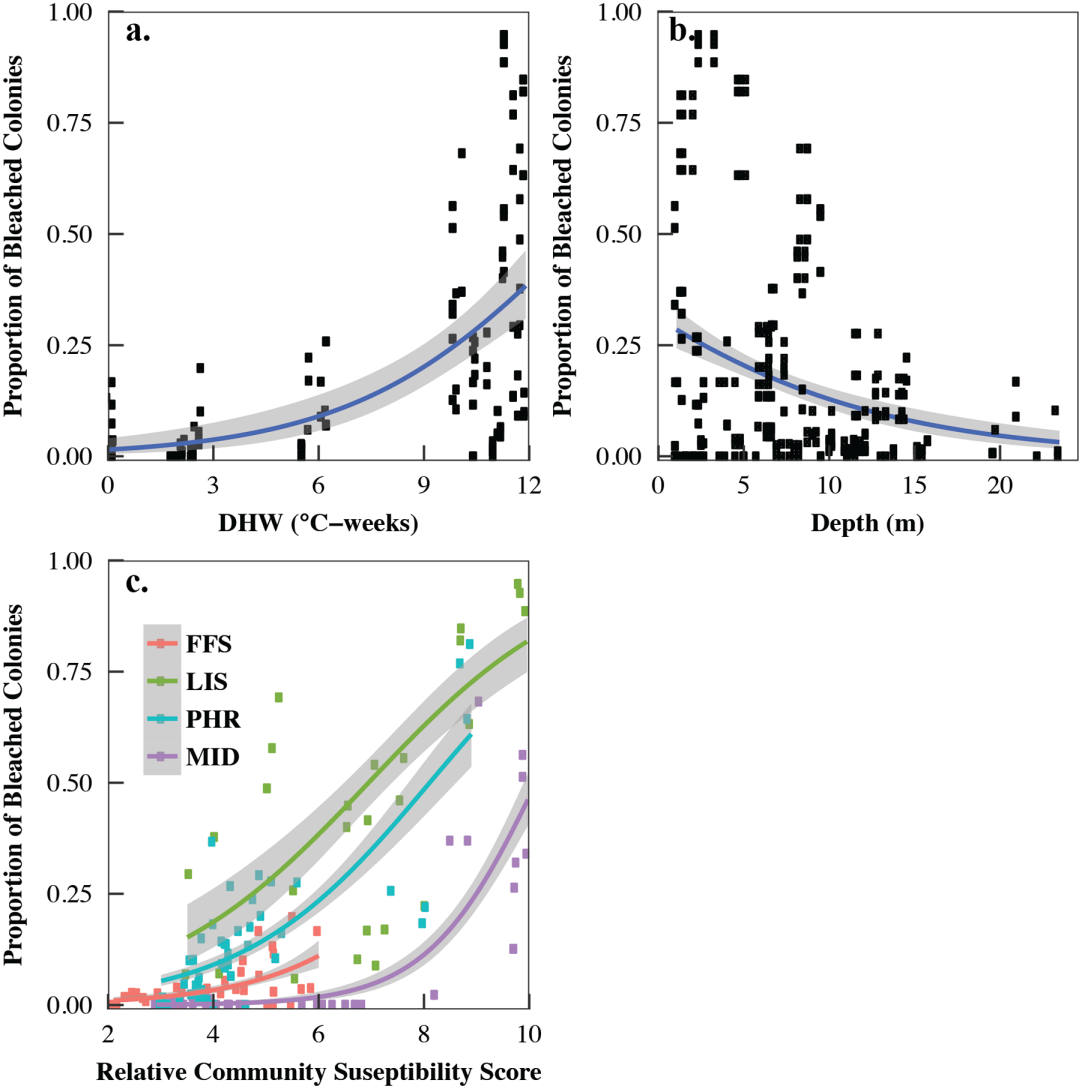
Generalized regressions of relationship between the proportion of bleached colonies and (a) CRW’s 5 km Degree Heating Week (DHW) during survey, (b) depth, and (c) the interaction of region and relative community susceptibility. Solid line: predicted bleaching (with binomial errors). Grey area: upper and lower 95% confidence intervals.

Percent bleaching in 2014 was best predicted by a combination of region, 5 km Degree Heating Week, relative community susceptibility score, and depth, which were included in the top three models with a cumulative AICc weight of 0.99 (Fig 6, Table 1, S4 Table). DHW and community susceptibility were both positively correlated with % bleaching and together explained 33% of the variance in bleaching (Fig 6a and 6b, Table 1). While depth was negatively correlated with % bleaching and was included in both of the top models, alone it only explained 2% of the variance in bleaching (Fig 6, Table 1). Region was also included in the best-fit models; however, when modeled alone it only explained 21% of the variance and had a delta AICc of 119.8 (Table 1, S4 Table). Interestingly, when we considered the relationship between region and community susceptibility, which was also included in all of the top models and accounted for 41% of the variance, we saw that corals at FFS had a lower susceptibility than other regions and bleaching increased more sharply with community susceptibility at LIS compared to MID (Fig 6c). When assessing the interaction between DHW and region, we saw that corals at PHR bleached less than LIS at high-levels of heat stress (>9°C-weeks) (S2 Fig).

**Table 1:** Model selection of GLMMs for % bleaching with region, CRW’s 5 km Degree Heating Week (DHW), depth, and relative coral community susceptibility score as fixed effects and site as a random effects. K: number of parameters in the model; AICc: the corrected Aikaike Information Criterion; ΔAICc: difference from the lowest AICc value; Marginal R^2^: proportion of total variance explained by the fixed effects alone; Conditional R^2^: proportion of the total variance explained by both the random and fixed effects. Model rank based on ΔAICc, with ΔAICc <10 indicating most reasonable models.

Rank	Fixed Effect Parameters	K	AICc	Δ AICc	AICc Wt	Marginal R^2^ (Fixed Effects)	Conditional R^2^ (Fixed & Random Effects)
1	DHW + Depth + Region x Susceptibility	11	2119.25	0.00	0.59	0.50	0.57
2	DHW + Region x Susceptibility	10	2120.94	1.70	0.25	0.49	0.57
3	Region x DHW + Depth + Region x Susceptibility	14	2122.02	2.78	0.15	0.51	0.57
4	Region + Depth + DHW + Susceptibility	8	2129.42	10.17	0.00	0.49	0.57
5	Region x DHW + Depth + Region + Susceptibility	11	2132.22	12.98	0.00	0.50	0.57
6	Region x DHW + Depth + Susceptibility	11	2132.22	12.98	0.00	0.50	0.57
7	Depth + Region x Susceptibility	10	2140.56	21.31	0.00	0.43	0.56
8	Region x Susceptibility	9	2142.72	23.47	0.00	0.41	0.57
9	Region + Depth + Susceptibility	7	2146.82	27.57	0.00	0.41	0.54
10	Region + Susceptibility	6	2154.31	35.06	0.00	0.34	0.52
11	DHW + Depth + Susceptibility	5	2158.58	39.34	0.00	0.35	0.54
12	DHW + Susceptibility	4	2158.72	39.47	0.00	0.33	0.54
13	Depth + Susceptibility	4	2181.44	62.19	0.00	0.17	0.51
14	Susceptibility	3	2181.49	62.25	0.00	0.13	0.50
15	Region x DHW + Depth	10	2208.86	89.61	0.00	0.44	0.59
16	Region x DHW	9	2209.72	90.48	0.00	0.45	0.61
17	Region + DHW	6	2216.12	96.88	0.00	0.39	0.60
18	Region + Depth	6	2229.18	109.93	0.00	0.28	0.55
19	DHW	3	2236.19	116.94	0.00	0.22	0.58
20	Region	5	2239.02	119.78	0.00	0.21	0.58
21	Depth	3	2256.30	137.05	0.00	0.02	0.57

### Implications of 2014 event on coral communities

**Fig 7:**
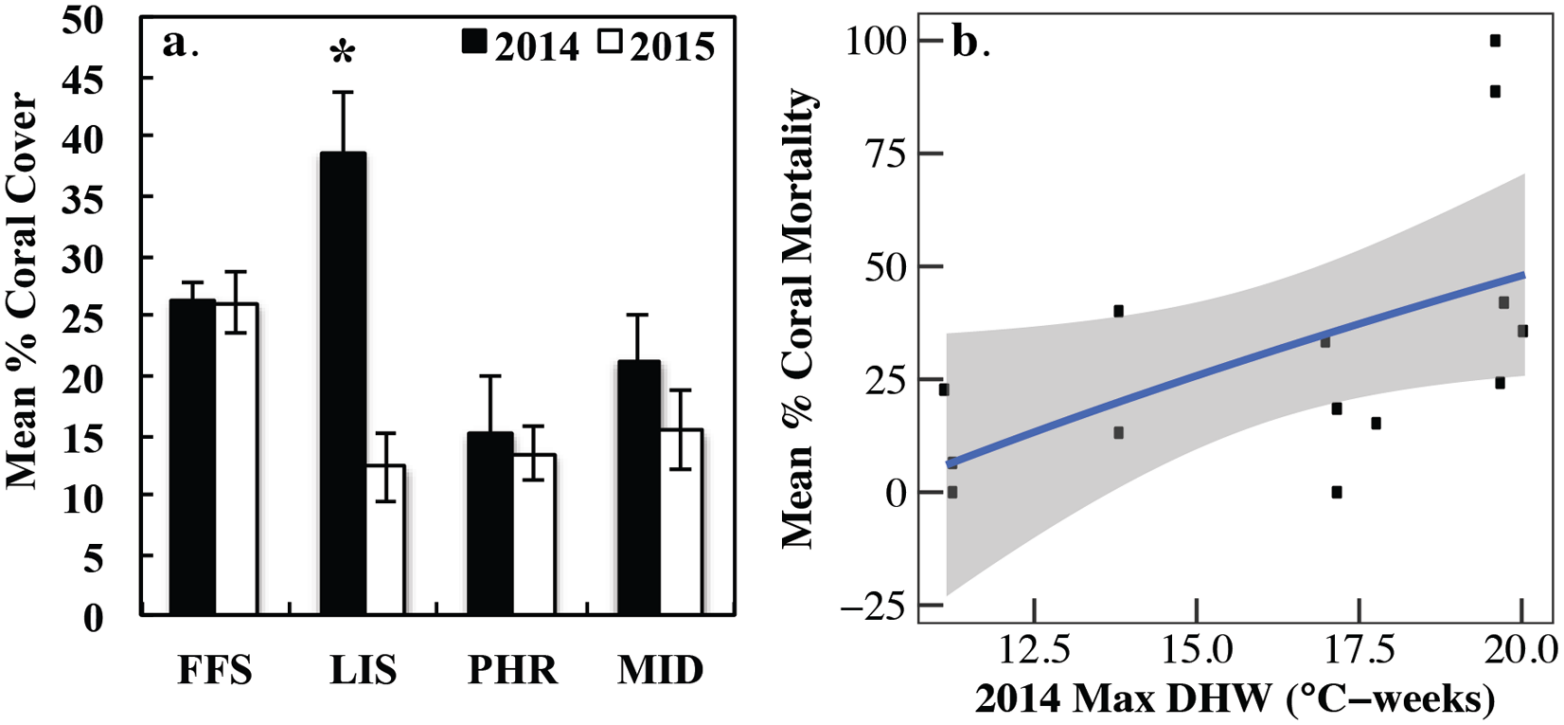
(a) Mean ± SE coral cover at permanent sites in September 2014 and August 2015. N = 12–18 transects per region/year. Asterisk indicates significant difference between years for each region (α = 0.0125). FFS = French Frigate Shoals, LIS = Lisianski Island, PHR = Pearl and Hermes Atoll, MID = Midway Atoll. (b) Mean % coral mortality (2014–2015% coral cover/2014% coral cover * 100) plotted against CRW’s maximum 5 km Degree Heating Weeks in 2014. Linear Regression: R^2^ = 0.3151, p = 0.0367.

Overall % coral cover decreased significantly in 2015 (LRT: χ^2^ = 7.63, df = 1, p = 0.0057) but also varied between regions (LRT: χ^2^ = 9.26, df = 3, p = 0.0257), with a significant region * year interaction (LRT: χ^2^ = 34.34, df = 7, p<0.0001) (Fig 7a). At the regional level, we only detected significant changes in mean % coral cover at LIS, which decreased by 68% (Fig 7a, LMM: ß = 3.07 ± 0.63, t = 4.87, p<0.0001), with a 35% to 77% loss in absolute coral cover along the eastern coast of LIS, equating to 89% to 100% decline on coral. Mean % coral cover did not change significantly at FFS (Fig 7a, LMM ß = 0.04 ± 0.30, t = 0.12, p = 0.905), PHR (Fig 7a, LMM ß = 0.28 ± 0.63, t = 0.44, p = 0.662) or MID (Fig 7a, LMM ß = 0.45 ± 0.75, t = 0.59, p = 0.552), although MID declined by 27%. The percent change in coral cover at LIS was strongly positively correlated with 2014 bleaching incidence (Linear Regression: R^2^ = 0.7929, p<0.0001). Change in cover was significantly and positively correlated with maximum DHW (Fig 7b).

Bleaching-related mortality at the species-level varied considerably between regions. While several *Montipora* spp. experienced mortality during 2014, M. *dilatata* complex, found in shallow habitats in LIS and back reefs at PHR and MID, was by far the most affected taxon, experiencing 100% mortality at both LIS and PHR, while remaining largely unchanged at MID (S5 Table).

### Changes in 3D reef structure

**Fig 8:**
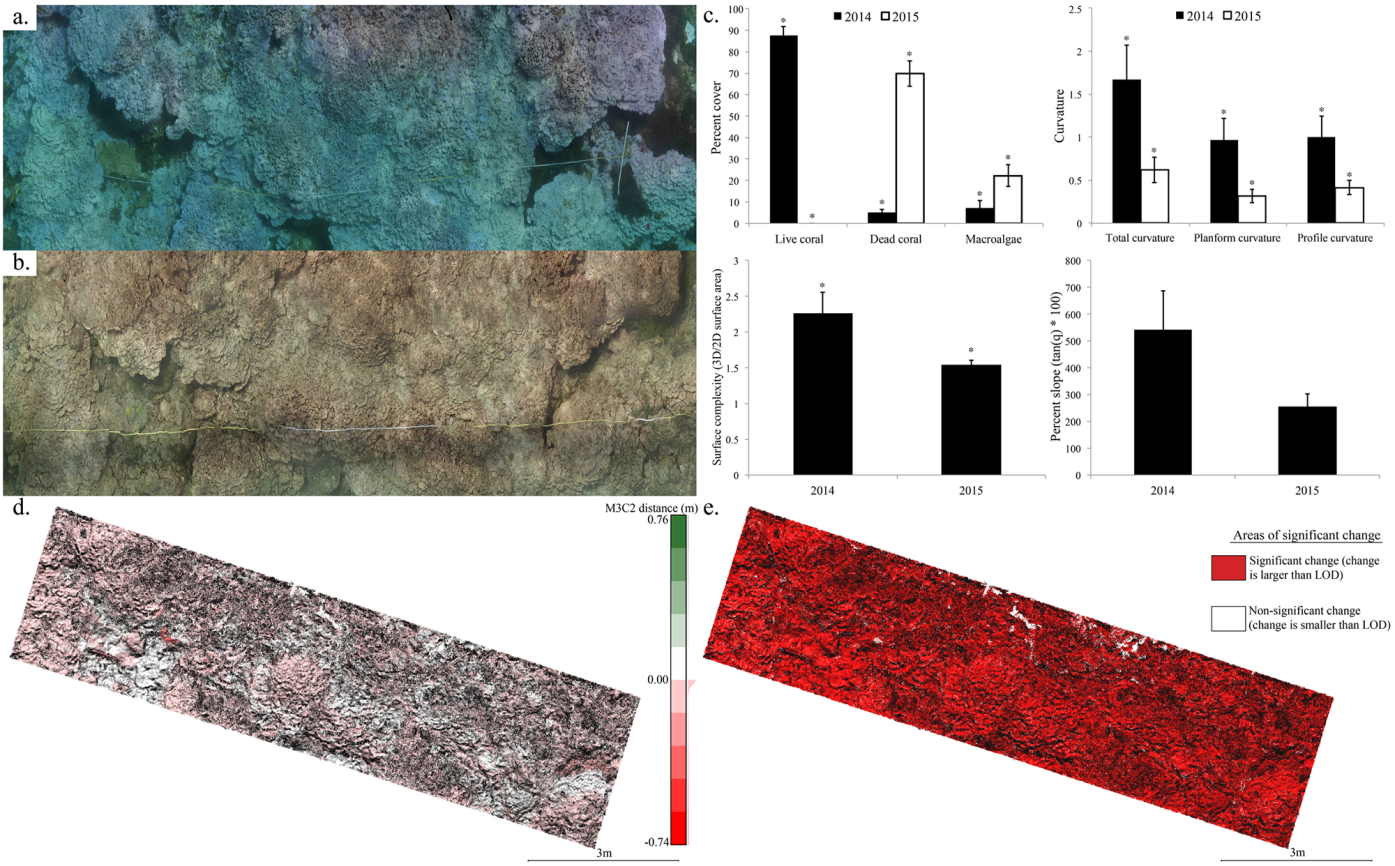
Orthophoto mosaics (10 x 3-m) representing the coral reef habitat at LIS-4067 (a) before (August 2014) and (b) after the bleaching event (August 2015). (c) Mean values (± SE) of community composition and 3D structural metrics before and after the bleaching event at Lisianski. Asterisk denotes significant differences (two sample t-test, p<0.05). The M3C2 algorithm shows measured change (d) in habitat volume as loss (red) and gain (green) for the reef substrate compared between the 3D point clouds from 2014 and 2015. The point cloud comparison also shows areas of significant 3D structural change (E) that were greater than the 95% level of detection (red).

At the most severely bleached site (LIS-4067) mean values of community composition and 3D structural metrics changed significantly following the bleaching event (Fig 8). The percent cover of live coral was significantly greater in 2014, and dead coral and macroalgae significantly greater in 2015 (two sample t-test, t = -9.25–34.78, p<0.05). Surface complexity, total curvature, planform, and profile curvature were all significantly lower in 2015 than in 2014 (two sample t-test, t = 2.37–3.10, p<0.05). Values of percent slope decreased after the bleaching, but not significantly (two sample t-test, t = -1.86, p = 0.10).

Fig 8d shows the areas of the reef substrate where habitat volume either increased (green) or decreased (red) after the bleaching event. The volumetric computation quantified a net loss in habitat volume of 1.496 m^3^in a 10 x 3-m plot. The early stage of declining structural complexity was also confirmed visually in 2015.

## Discussion

### Long-term change in heat stress

Long-term satellite data provides preliminary support that the scale and magnitude of heat stress may be changing in PMNM—as it has for reefs around the world. Since 1982, there have been eight bleaching-level heat stress events (≥ 4°C-weeks) observed in PMNM, with bleaching reported in seven of the eight heat stress events (Fig 2, this study, Vargas-Ángel pers. comm.). These long-term data underscore how unique the 2014 event was with up to 19.69°C-weeks in 2014 and the entire PMNM exposed to mortality-level heat stress 2014 (Fig 1). Changing heat stress is also being felt throughout the archipelago with many of the Main Hawaiian Islands experiencing bleaching for the first time in reported history [49, 64] and some regions experiencing unprecedented consecutive years of bleaching [65, Ruiz-Williams pers. comm., Hawaiʻi DAR pers. comm., Massey pers. comm.]. Our study adds to the growing number of studies reporting significant increases in heat stress over the last 30 to 60 years [2, 7, 11, 31]. For example, Heron et al. [31] found that 97% of reefs globally are warming, with an elongation of summer seasons at most locations, resulting in a four to five fold increase in bleaching-level heat stress (≥ 4°C-weeks) between 1982 and 2012 in the Middle East and Atlantic basins. The recent bleaching events in the Hawaiian Archipelago may presage arrival of a new norm just as the record global temperatures in 2014, 2015, and 2016 have driven the first 3-year global coral bleaching event ever seen. Unfortunately, 2017 temperatures have continued to be among the hottest on record (NOAA-NCEI annual climate reports, https://www.ncdc.noaa.gov/sotc/global) with continued bleaching including the first recorded back-to-back bleaching on the Great Barrier Reef in 2016 and 2017 [11].

### Heat stress and coral community drivers of 2014 bleaching

In 2014, bleaching sensitivity varied considerably likely due to a complex series of factors. Consistent with previous studies [1, 46, 47, 59], we found that Degree Heating Week explained up to 22% of the variance in bleaching alone and 45% when combined with region (Table 1). However, there was a high degree of variability in bleaching in reefs experiencing greater than 9°C-weeks suggesting that other factors such as taxon-specific susceptibility need to be considered alongside heat stress. While satellite-derived heat stress data are widely used to explain past and forecast future bleaching [1, 37, 66, 67], *in situ* data would likely improve its predictive power especially on deeper reefs and reefs exposed to subsurface waves.

Consistent with previous studies, bleaching was also strongly mediated by the distribution of susceptible species [19, 34, 47, 59, 60, 68–70]. Overall, in 2014, relative community susceptibility explained 13% of the variance in bleaching alone (Table 1). Following the bleaching hierarchy reported in other regions [19, 47, 59, 68–70], the highly susceptible communities were dominated by certain taxa in the families Acroporidae and Pocilloporidae, especially *Montipora* spp., with 50% of *Montipora dilatata* complex bleaching across all study sites and up to 94% at LIS (S3 Fig, S3 Table). This species’ high sensitivity has been reported on other Hawaiian reefs and was the first species to bleach during the 1996 bleaching event in Kāne‘ohe Bay [2]. Unlike other Indo-Pacific reefs, where *Acropora* are often the most susceptible to bleaching [68] [20, 59, 70], we observed very low bleaching in this taxon, which is consistent with some reefs in Southeast Asia [34]. With almost all of PMNM’s *Acropora* found at FFS (primarily *Acropora cytherea*), the low bleaching is likely attributed to low heat stress at FFS, which did not exceed 6°C-weeks during our 2014 surveys. However, FFS did experience an unprecedented 10.81°C-weeks in 2014 (Fig 2c). While quantitative coral cover assessments could not be conducted in 2015 at *Acropora*-dominated sites due to high currents, visual assessments in 2015 confirmed that these reefs did not experience major mortality suggesting that these populations may be more resilient to bleaching than other *Acropora*-dominated Indo-Pacific reefs. Other regional factors may have also influenced community susceptibility. For example, MID experienced lower bleaching in communities with high susceptibility compared to LIS (Fig 6c). While this pattern may be explained by differences in heat stress, it is unlikely given that average heat stress during the surveys only varied by 1°C-week (LIS: 8.67°C-weeks, MID: 7.72°C-weeks).

In addition to the magnitude of accumulated heat stress and community susceptibility, other environmental factors may be driving bleaching across PMNM. While depth alone only explained 1% of the variance, it was important in context with the other variables. The relationship between bleaching and depth can vary considerably depending on a suite of factors, but consistent with other regions [58, 59, 71], decreased bleaching with depth suggests that changes in community structure, subsurface waves, thermocline shoaling, and/or general patterns of lower irradiance and temperature on deeper reefs may have influenced bleaching susceptibility. Regional differences in bleaching may also be explained by variation in irradiance and turbidity, whereby corals in clearer water with higher irradiance bleached more [2, 39–41]. Regional differences in irradiance and turbidity seem less likely given that the most severely bleached region (LIS) was also the most turbid during 2014 surveys (Couch pers. obs). The low winds associated with 2014 and previous bleaching events lead to reduced water circulation and vertical mixing, especially in sheltered habitats, may have also exacerbated coral physiological stress and bleaching susceptibility [2]. Alternatively, the narrow reef shelves and surrounding oceanography in the northern atolls may have enhanced water circulation on fore reefs and hence reduced heat stress at PHR and MID compared to the wider shallow shelf at LIS. Thermal variability can also affect bleaching susceptibility whereby reefs that have experienced prior exposure to higher thermal variability are physiologically conditioned to handle heat stress [43–45]. PMNM is particularly unique in that the northern atolls experience some of the highest temperature variability of any global coral reef with 6–7°C annual temperature fluctuation [72], with certain fore reef sites at PHR fluctuating 6°C daily [72] due to internal waves and upwelling [73]. While SST variability from satellite data increases with latitude (S1 File), there was not enough within-region variability to include in our models, but should be investigated using subsurface temperature loggers.

### Potential signs of acclimation

There is considerable debate over the capacity of coral reefs to acclimatize and adapt to the mounting heat stress and repeated bleaching events with some finding no evidence of acclimation or adaptation [11] and others finding reduced bleaching and mortality on reefs exposed to previous heat stress [34, 45, 70, 74, 75]. In PMNM, average heat stress was up to 8.5 times higher in 2014 than previous years (Figs 1 and 2, S2 Table), but % bleaching at PHR and MID was higher in 2002 compared to 2004 and 2014. Corals across all the permanent sites also bleached more at lower heat stress in 2002 and 2004 compared to 2014 (Fig 3b). This suggests that bleaching sensitivity was indeed highest during early exposure to heat stress levels. After correcting for temporal differences in heat stress, PHR bleached more in 2002 than expected given heat stress (Fig 3c), suggesting that coral communities may have shifted towards a less susceptible community following earlier bleaching events as seen in other studies [19–21]. On the back reefs at PHR and MID, the 2002 bleaching event resulted in coral mortality and subsequent proliferation of macroalgae (Vroom and Kenyon pers. comm., [49]). However, when we compared species composition between years we did not detect a significant shift in the community structure at PHR (Fig 3d). While changes in community composition were not tested at MID, the communities were largely comprised of *M*. *dilatata* complex and *M*. *capitata* in all three years. Alternatively, this suggests that prior to 2014 there had been some level of acclimation at PHR and possibly the back reefs at MID. Recent studies from Kāne‘ohe Bay suggest that repeated bleaching may lead to changes in bleaching resistance and susceptibility. For example, unlike the 1996 event, during 2014 *M*. *capitata* was more resistant than the typically hardy *P*. *compressa* [51]. While molecular and physiological assessments are needed to confirm whether these northern atolls are showing signs of acclimation or adaptation, improved resilience following serial heat stress and bleaching may be attributed to shifts in their *Symbiodinium* populations [76–79], epigenetic changes turning on heat shock and related proteins in the corals [80, 81] and/or selection of resilient coral genotypes [82, 83].

### Implications of 2014 event on coral communities

While assessing bleaching patterns is necessary for predicting future bleaching resilience, quantifying mortality and bleaching recovery are even more ecologically important. Indices that combine bleaching and mortality [34, 47, 84] are highly dependent on the timing of the surveys in relation to the amount of heat stress, thus repeat observations are needed for accurate assessments of mortality. We found that coral communities varied widely in their capacity to recover with dramatic changes to coral community composition and 3D reef structure in regions such as LIS which experienced a 68% decline in coral cover. Community-level recovery may be explained by a combination of differences in community structure and local resilience. For example, 89–99% of coral along the eastern coast of Lisianski Island consisted of the most sensitive taxon, *Montipora dilatata* complex, which suffered severe morality in 2014. While mortality still occurred at other LIS sites, southerly *Porites*-dominated sites demonstrated lower mortality and greater capacity for recovery from bleaching. Despite not seeing significant coral mortality at PHR in 2014, the small *M*. *dilatata* complex population at the back reef sites disappeared, while the other species remained unchanged. The MID back reef was a different story with a 27%, albeit non-significant, decline in *M*. *capitata*, but minimal mortality in the abundant *M*. *dilatata* complex, which bleached severely during 2002 and 2004. Similar to other locations [20, 34, 46–48], these data together with the bleaching data suggest that there are some winners and losers in terms of species in PMNM, but this may vary across islands and atolls and through time.

### Changes in structural complexity following 2014 event

Our 3D reef reconstructions demonstrated that in addition to severe coral mortality, bleaching is also significantly reducing habitat complexity on time scales much faster than originally believed (Fig 8c). The loss of structural complexity across 99% of the area of the most severely bleached site (Fig 8d and 8e) is substantial when compared to partially bleached sites in the Main Hawaiian Islands, which only exhibited 30–40% area of significant structural change using similar methods [29]. Interestingly, Jarvis Island in the central Equatorial Pacific experienced >95% coral morality following the 2015 bleaching event [85], which resulted in similar widespread and visible loss in structural complexity by 2016 (Vargas-Ángel pers. obs.). In prior reports of mass bleaching, breakdown of the reef structure is typically measurable in 3 to 5 years using conventional metrics for habitat complexity [21–25]. This differs from our findings and observations from other Pacific reefs that detected quantitative or qualitative changes in one year and may be due to a number of factors. First, the most common methods for assessing reef complexity have been the chain method or visual estimates [21–25, 27, 86]. However, those methods provide limited spatial coverage across a reef and a crude measure compared to high-resolution SfM 3D reconstruction techniques. Second, the severity of recent mortality exposed 90–100% of the underlying carbonate structure to bioeroders, which often increases following bleaching events [87]. In addition, the loss of almost all of the live tissue also reduced coral accretion to near zero. Finally, as mentioned by Pratchett et al. [22], the robustness of a reef structure can depend on the species composition with reefs dominated by faster growing taxa such as *Acropora* less robust to disturbances. In the case of eastern Lisianski Island and Jarvis Island, these regions were dominated by highly susceptible and fragile *Montipora* spp. prior to the bleaching events thus exacerbating reef erosion. In addition to highlighting the value of using our SfM 3D reconstruction techniques, our findings also emphasize the potential implications of this and future bleaching events to alter ecosystem function through losses of habitat space, biodiversity, and organismal abundance, especially in communities dominated by fast growing fragile taxa.

### Role of climatic processes in heat stress across Hawaiʻi

When elucidating current and future susceptibility to bleaching, it is important to consider the climatic processes driving heat stress. The climatic processes underpinning Hawaiʻi’s recent bleaching events are especially complex and involve climate change, “The Blob”, Pacific Decadal Oscillation, and El Niño. In Hawai‘i, a significant long-term warming trend was first documented in O‘ahu’s, offshore waters by Jokiel and Coles [41] and Midway Atoll [2] and has been attributed to climate change through the anthropogenic production of CO_2_. Through an extension of these earlier analyses, Bahr et al. (2015) confirmed that warming has continued offshore O‘ahu with a 1.5°C increase during the last 58 years. An unusually large mass of warm water (nicknamed “The Blob”, [88]), which originated in the Pacific Ocean off the northwestern coast of North America, has had direct connection with the warming in the Hawaiian Islands as revealed in satellite data. The Blob was first detected in fall 2013, and was associated with temperatures 2.5°C above normal in February 2014 [88]. North Pacific warming associated with The Blob resulted in a spread of heat stress across the PMNM by summer 2014. While the Blob did not dissipate until January 2016, it appeared to play less of role in the 2015 warming in Hawaiʻi. Based on dOISST.v2 data, previous heat stress, including during the 2002 event in PMNM, may have been linked to similar patterns of North Pacific warming (Fig 1). Previous studies have also hypothesized that recent bleaching events may be linked to the Pacific Decadal Oscillation (PDO), which affects Northern Pacific regions including Hawai‛i [2, 51]. During the last century, PDO has fluctuated through cool and warm phases every 15 to 25 years and 50 to 70 years [89]. In 2014, the PDO index reached its highest and hottest value in recorded history and remained high throughout 2015 (Joint Institute for the study of Atmosphere and Ocean, 2016), leading Bahr et al. [51] to hypothesize that PDO fluctuations may have played a role in the 2014 and 2015 events. The 2015–2016 ENSO event contributed to the third reported global bleaching event for most reefs worldwide [36]. However, unlike most tropical waters, ocean temperatures on Hawaiian reefs are generally not correlated with ENSO and none of the prior bleaching events in Hawaiʻi occurred during ENSO events [e.g. 2, 4, 7]. This does not eliminate the possibility, though, that the 2015–16 El Niño may have interacted with other climatic process to exacerbate 2015 warming as conditions during ENSO events can vary greatly.

## Conclusions

The mounting intensity of mass coral bleaching events and the unprecedented three consecutive years of global bleaching are reshaping many of the world’s reefs. As evidenced by a wealth of studies and observations, this is strongly associated with climate change and continuous warming of the oceans. This punctuates the need for significant reduction in the atmospheric concentration of CO_2_ as the main contributor to the rapidly ongoing climate change as well as continued monitoring. Physiological assessments and community-level monitoring will allow us to not only identify physiological characteristics important for resilience, but also determine how naïve or previously bleached reefs respond to increasing heat stress. Together these assessments will help us to understand how stressed coral reef ecosystems may recover both in the short and long term. Through this process we will also be able to refine our prediction capability to pinpoint both susceptible and resilient coral reef systems. Our study highlights that interpreting the complex patterns in bleaching and recovery/mortality needs to go beyond just assessing heat stress and requires an understanding of community susceptibility, local habitat characteristics, and thermal history. Our study indicates that novel severe heat stress at Lisianski may have led to significant mortality in 2014. In contrast, corals in regions such as Pearl and Hermes and Midway, which experienced prior heat stress, bleached less in 2014 than in 2002 –despite higher heat stress in 2014. The changes in coral community composition at Lisianski following the 2014 bleaching may influence susceptibility to future heat stress. In fact, *Montipora dilatata* complex, one of the historically dominant but thermally sensitive reef-building species, was showing promising recovery by summer 2015 at Midway. PMNM’s capacity to resist and recover from future bleaching will likely be dependent on factors such as the duration between events and recovery time, acclimatization of symbiont communities and coral hosts, and recruitment and propagation of bleaching tolerant genotypes. Despite being largely free from the local stressors that face many reefs, the reefs of PMNM are still subject to rising ocean temperature and acidification. Lacking local stressors, protected remote reef areas such as PMNM will provide key information on whether corals and coral reefs can respond quickly enough to survive the rapid rise of anthropogenic carbon dioxide and other heat-trapping gases and the degree to which local disturbances influence bleaching resilience

## Supporting information

S1 Table Parameter estimates for GLM (binomial errors and logit link) of % bleaching in 2002, 2004 and 2014 vs. Degree Heating Week (DHW). **Bold** indicates significant effect of covariate at p<0.05. DHW is centered and scaled.
(DOCX)

S2 Table Heat stress at study sites during time of surveys and annual maximum. Survey dates for each region, mean Degree Heating Weeks ± SE at the time of survey and annual maximum degree heating weeks across different regions in 2002, 2004, and 2014 for the CRW’s dOISST.v2 data (1 pixel/site/day, n = 2–5 sites/region) and 2014 and 2015 for the CRW 5km data (1 pixel/site/day, n = 14–16 sites/region).
(DOCX)

S3 Table Bleaching patterns & susceptibility scores across species. Mean % bleaching (% of colonies that lost >50% of pigmentation) by species across all sites and regions in August and September 2014. All coral species were scored from least (1) to most susceptible (10) to bleaching using a combination of species bleaching levels in 2014 (S2 Table), 2004 [49] and personal communication with Bernardo Vargas-Ángel at NOAA PIFSC’s Ecosystem Sciences Division.
(DOCX)

S4 Table Parameter estimates for top 2 ‘best-fit’ GLMM models of 2014 bleaching. **Bold** indicates significant effect of covariate at p<0.05. Full list of models in Table 1.
(DOCX)

S5 Table Changes in % cover of individual species across four regions following 2014 bleaching event. Mean ± SE % cover by region and year for species with >1% cover on a given transect. N = 6–12 transects/habitat/region. FFS = French Frigate Shoals, LIS = Lisianski Island, PHR = Pearl and Hermes Atoll, MID = Midway Atoll. Asterisk indicates significant difference between years after Bonferroni correction.
(DOCX)

S1 Fig Binomial regression of the relationship between predicted proportion of bleached coral (% bleaching) in 2002, 2004, and 2014 and Degree Heating Week. Generalized regressions of relationship between the proportion of bleached colonies and DHW during survey based on CRW’s dOISST.v2 dataset. Solid line: predicted bleaching (with binomial errors). Grey area: upper and lower 95% confidence intervals.
(DOCX)

S2 Fig Binomial regressions of the relationship between predicted proportion of bleached coral (% bleaching) in 2014 and Region x DHW. Generalized regressions of relationship between the proportion of bleached colonies the interaction of region and degree heating week (DHW). Solid lines: predicted bleaching (with binomial errors) for each region. Grey area: upper and lower 95% confidence intervals.
(DOCX)

S3 Fig Bleaching patterns across species and region. Mean % bleaching summarized by region and species for species with >1% cover on a given transect. FFS = French Frigate Shoals, LIS = Lisianski Island, PHR = Pearl and Hermes Atoll, MID = Midway Atoll.
(DOCX)

S4 Fig Bleaching at permanent sites in 2014 and 2015. (a) Mean ± SE bleaching at permanent sites in September 2014 and August 2015. N = 12–18 transects per region/year. Asterisk indicates significant difference between years for each region (α = 0.0125). FFS = French Frigate Shoals, LIS = Lisianski Island, PHR = Pearl and Hermes Atoll, MID = Midway Atoll.
(DOCX)

S1 File File includes metadata for all sites including latitude, longitude, depth, and site name. It also includes % bleaching data for 2002, 2004, 2014 and 2015 at the site and species level, as well as % coral cover data for 2014 and 2015 at the species and site level. File also includes all CRW 5kmv3 and dOISST.v2 data for each site.
(ZIP)

S2 File File includes topographic data extracted from the orthophotomosaic and digital elevation models using ArcMap geospatial software for LIS_4067 in 2014 and 2015. (XLSX)
