# Supplementary material for: Mass coral bleaching due to unprecedented marine heatwave in Papahānaumokuākea Marine National Monument (Northwestern Hawaiian Islands)
Source: PLoS One. 2017 Sep 27;12(9):e0185121. doi: 10.1371/journal.pone.0185121 (PMC5617177; doi:10.1371/journal.pone.0185121)
Supplement: S1 Table — Bold indicates significant effect of covariate at p<0.05. DHW is centered and scaled. (DOCX) [file pone.0185121.s001.docx]

**S1. Table.** **Parameter estimates for GLM (binomial errors and logit link) of % bleaching in 2002, 2004 and 2014 vs. degree heating week (DHW)**. **Bold** indicates significant effect of covariate at p<0.05. DHW is centered and scaled.
