# Supplementary material for: Mass coral bleaching due to unprecedented marine heatwave in Papahānaumokuākea Marine National Monument (Northwestern Hawaiian Islands)
Source: PLoS One. 2017 Sep 27;12(9):e0185121. doi: 10.1371/journal.pone.0185121 (PMC5617177; doi:10.1371/journal.pone.0185121)
Supplement: S2 Table — Survey dates for each region, mean Degree Heating Weeks ± SE at the time of survey and annual maximum degree heating weeks across different regions in 2002, 2004, and 2014 for the CRW’s dOISST.v2 data (1 pixel/site/day, n = 2–5 sites/region) and 2014 and 2015 for the CRW 5km data (1 pixel/site/day, n = 14–16 sites/region). (DOCX) [file pone.0185121.s002.docx]

**S2 Table**. **Heat stress at study sites during time of surveys and annual maximum.** Survey dates for each region, mean degree heating weeks ± SE at the time of survey and annual maximum degree heating weeks across different regions in 2002, 2004, and 2014 for the dOISST.v2 data (1 pixel/site/day, n = 2-5 sites/region) and 2014 and 2015 for the CRW 5kmV3 data (1 pixel/site/day, n = 14-16 sites/region).
