# Supplementary material for: Mass coral bleaching due to unprecedented marine heatwave in Papahānaumokuākea Marine National Monument (Northwestern Hawaiian Islands)
Source: PLoS One. 2017 Sep 27;12(9):e0185121. doi: 10.1371/journal.pone.0185121 (PMC5617177; doi:10.1371/journal.pone.0185121)
Supplement: S3 Table — Mean % bleaching (% of colonies that lost >50% of pigmentation) by species across all sites and regions in August and September 2014. All coral species were scored from least (1) to most susceptible (10) to bleaching using a combination of species bleaching levels in 2014 (S2 Table), 2004 [49] and personal communication with Bernardo Vargas-Ángel at NOAA PIFSC’s Ecosystem Sciences Division. (DOCX) [file pone.0185121.s003.docx]

**S3 Table.** **Bleaching patterns & susceptibility scores across species.** Mean % bleaching (% of colonies that lost >50% of pigmentation) by species across all sites and regions in August and September 2014. All coral species were scored from least (1) to most susceptible (10) to bleaching using a combination of species bleaching levels in 2014 (S2 Table), 2004 (Kenyon et al. 2006) and unpublished data from NOAA PIFSC’s Ecosystem Sciences Division.
