# Supplementary material for: Mass coral bleaching due to unprecedented marine heatwave in Papahānaumokuākea Marine National Monument (Northwestern Hawaiian Islands)
Source: PLoS One. 2017 Sep 27;12(9):e0185121. doi: 10.1371/journal.pone.0185121 (PMC5617177; doi:10.1371/journal.pone.0185121)
Supplement: S4 Table — Bold indicates significant effect of covariate at p<0.05. Full list of models in Table 1. (DOCX) [file pone.0185121.s004.docx]

**S4 Table.** **Parameter estimates for top 2 ‘best-fit’ GLMM models of 2014 bleaching**. **Bold** indicates significant effect of covariate at p<0.05. Full list of models in Table 1.
