# Supplementary material for: Mass coral bleaching due to unprecedented marine heatwave in Papahānaumokuākea Marine National Monument (Northwestern Hawaiian Islands)
Source: PLoS One. 2017 Sep 27;12(9):e0185121. doi: 10.1371/journal.pone.0185121 (PMC5617177; doi:10.1371/journal.pone.0185121)
Supplement: S5 Table — Mean ± SE % cover by region and year for species with >1% cover on a given transect. N = 6–12 transects/habitat/region. FFS = French Frigate Shoals, LIS = Lisianski Island, PHR = Pearl and Hermes Atoll, MID = Midway Atoll. Asterisk indicates significant difference between years after Bonferroni correction. (DOCX) [file pone.0185121.s005.docx]

**S5 Table. Changes in % cover of individual species across four regions following the 2014 bleaching event.** Mean ± SE % cover by region and year for species with >1% cover on a given transect. N= 6-12 transects/ habitat/ region. FFS= French Frigate Shoals, LIS = Lisianski Island, PHR = Pearl and Hermes Atoll, MID = Midway Atoll. Asterisk indicates significant difference between years after Bonferroni correction.
