# Supplementary material for: Mass coral bleaching due to unprecedented marine heatwave in Papahānaumokuākea Marine National Monument (Northwestern Hawaiian Islands)
Source: PLoS One. 2017 Sep 27;12(9):e0185121. doi: 10.1371/journal.pone.0185121 (PMC5617177; doi:10.1371/journal.pone.0185121)
Supplement: S1 Fig — Generalized regressions of relationship between the proportion of bleached colonies and DHW during survey based on CRW’s dOISST.v2 dataset. Solid line: predicted bleaching (with binomial errors). Grey area: upper and lower 95% confidence intervals. (DOCX) [file pone.0185121.s006.docx]

**S1 Figure. Binomial regression of the relationship between predicted proportion of bleached coral (% bleaching) in 2002, 2004, and 2014 and degree heating week.** Generalized regressions of relationship between the proportion of bleached colonies and DHW during survey. Solid line: predicted bleaching (with binomial errors). Grey area: upper and lower 95% confidence intervals.
