# Supplementary material for: Mass coral bleaching due to unprecedented marine heatwave in Papahānaumokuākea Marine National Monument (Northwestern Hawaiian Islands)
Source: PLoS One. 2017 Sep 27;12(9):e0185121. doi: 10.1371/journal.pone.0185121 (PMC5617177; doi:10.1371/journal.pone.0185121)
Supplement: S2 Fig — Generalized regressions of relationship between the proportion of bleached colonies the interaction of region and degree heating week (DHW). Solid lines: predicted bleaching (with binomial errors) for each region. Grey area: upper and lower 95% confidence intervals. (DOCX) [file pone.0185121.s007.docx]

**S2 Figure. Binomial regressions of the relationship between predicted proportion of bleached coral (% bleaching) in 2014 and Region x DHW.** Generalized regressions of relationship between the proportion of bleached colonies the interaction of region and degree heating week (DHW). Solid lines: predicted bleaching (with binomial errors) for each region. Grey area: upper and lower 95% confidence intervals.
