# Supplementary material for: Mass coral bleaching due to unprecedented marine heatwave in Papahānaumokuākea Marine National Monument (Northwestern Hawaiian Islands)
Source: PLoS One. 2017 Sep 27;12(9):e0185121. doi: 10.1371/journal.pone.0185121 (PMC5617177; doi:10.1371/journal.pone.0185121)
Supplement: S3 Fig — Mean % bleaching summarized by region and species for species with >1% cover on a given transect. FFS = French Frigate Shoals, LIS = Lisianski Island, PHR = Pearl and Hermes Atoll, MID = Midway Atoll. (DOCX) [file pone.0185121.s008.docx]

**S3 Figure.** **Bleaching patterns across species and region.** Mean % bleaching summarized by region and species for species with >1% cover on a given transect. FFS= French Frigate Shoals, LIS = Lisianski Island, PHR = Pearl and Hermes Atoll, MID = Midway Atoll.
