# Supplementary material for: Mass coral bleaching due to unprecedented marine heatwave in Papahānaumokuākea Marine National Monument (Northwestern Hawaiian Islands)
Source: PLoS One. 2017 Sep 27;12(9):e0185121. doi: 10.1371/journal.pone.0185121 (PMC5617177; doi:10.1371/journal.pone.0185121)
Supplement: S4 Fig — (a) Mean ± SE bleaching at permanent sites in September 2014 and August 2015. N = 12–18 transects per region/year. Asterisk indicates significant difference between years for each region (α = 0.0125). FFS = French Frigate Shoals, LIS = Lisianski Island, PHR = Pearl and Hermes Atoll, MID = Midway Atoll. (DOCX) [file pone.0185121.s009.docx]

**S4 Figure. Bleaching at permanent sites in 2014 and 2015** (a) Mean ± SE bleaching at permanent sites in September 2014 and August 2015. N= 12-18 transects per region/year. Asterisk indicates significant difference between years for each region (α = 0.0125). FFS= French Frigate Shoals, LIS = Lisianski Island, PHR = Pearl and Hermes Atoll, MID = Midway Atoll.
